# Anti-Inflammatory Efficacy of Human-Derived *Streptococcus salivarius* on Periodontopathogen-Induced Inflammation

**DOI:** 10.4014/jmb.2302.02002

**Published:** 2023-05-11

**Authors:** Dong-Heon Baek, Sung-Hoon Lee

**Affiliations:** Department of Dental Hygiene, College of Health Science, Dankook University, Cheonan 31116, Republic of Korea

**Keywords:** *Streptococcus salivarius*, lipoteichoic acid, anti-inflammatory efficacy, periodontitis, Toll-like receptor

## Abstract

*Streptococcus salivarius* is a beneficial bacterium in oral cavity, and some strains of this bacterium are known to be probiotics. The purpose of this study was to investigate the anti-inflammatory effect and mechanism of *S. salivarius* G7 lipoteichoic acid (LTA) on lipopolysaccharide (LPS) and LTA of periodontopathogens. The surface molecules of *S. salivarius* G7 was extracted, and single- or co-treated on human monocytic cells with LPS and LTA of periodontopathogens. The induction of cytokine expression was evaluated by real-time PCR and ELISA. After labeling fluorescence on LPS and LTA of periodontopathogens, it was co-treated with *S. salivarius* LTA to the cell. The bound LPS and LTA were measured by a flow cytometer. Also, the biding assay of the LPS and LTA to CD14 and LPS binding protein (LBP) was performed. The surface molecules of *S. salivarius* G7 did not induce the expression of inflammatory cytokines, and *S. salivarius* G7 LTA inhibited the inflammatory cytokines induced by LPS and LTA of periodontopathogens. *S. salivarius* G7 LTA inhibited the binding of its LPS and LTA to cells. Also, *S. salivarius* G7 LTA blocked the binding of its LPS and LTA to CD14 and LBP. *S. salivarius* G7 has an inhibitory effect on inflammation induced by LPS or LTA of periodontopathogens, and may be a candidate probiotics for prevention of periodontitis.

## Introduction

The major bacteria related diseases in the oral cavity can be broadly divided into diseases of teeth and gingival region. Periodontitis, one of the diseases of the gingival region initiates host immune response of gingival tissue against virulence factors of Gram-negative anaerobe in oral cavity [1]. Among Gram-negative anaerobe, *Porphyromonas gingivalis* (*P. gingivalis*), *Tannerella forsythia* (*T. forsythia*), and *Treponema denticola* (*T. denticola*) were classified as a red complex by Socransky group because they were frequently detected in patient with periodontitis according to epidemiological examination [2]. Also, *Filifactor alocis* (*F. alocis*) is recently emerging as important bacterium related with periodontitis because this bacterium is detected in endodontic and periodontal inflammation regions [3, 4]. Furthermore, unlike other periodontopathogens, *F. alocis* is Gram-positive microbe and has a virulent factor as lipoteichoic acid (LTA) [5].

*Streptococcus salivarius* (*S. salivarius*) is an early colonizer species that colonizes the human oral cavity from newborn infant and persists a predominant member of the commensal microbiota throughout the life [6, 7]. Recently, *S. salivarius* is included in the inventory of microbial food cultures with safety demonstration in fermented food products according to the international Dairy Federation [8]. Many strains produce bacteriocin-like inhibitory substance (BLIS) which plays an important role in both stabilizing the oral microbiota and preventing overgrowth by various pathogens with infectious disease in oral cavity [9]. The antibacterial activity using BLIS on many strains of *S. salivarius* has been examined, and most *S. salivarius* showed antibacterial activity against periodontopathogens [10-12]. Also, the isolated strain K12 has been showed neutralizing halitosis and inhibition of biofilm formation [10, 13]. These characteristics of *S. salivarius* are being attempted to be developed as probiotics that maintain oral healthy, and many studies have shown results safety and antibacterial activity of isolated *S. salivarius* [13-16].

The effects of *S. salivarius* on periodontitis was presented as a result of its antibacterial activity against the related bacteria and the existing studies on biofilm. However, among the many studies of *S. salivarius*, the study on the inhibitory mechanism of *S. salivarius* for the induction of inflammation by lipopolysaccharide (LPS) of periodontopathogens is not little known.

The aim of present study was to be investigate the inhibitory effect of *S. salivarius* G7 LTA on inflammatory cytokines-induced by LPS or LTA of periodontopathogen and be analyze mechanism of their anti-inflammation.

## Materials and Methods

### Bacterial Species and Culture Conditions

Isolated strain of *Streptococcus salivarius* G7 (formerly KCOM 2137) from human oral cavity were used in this study and donated from Greenstore Co. (Korea). *S. salivarius* ATCC 7073 as a type strain was used as a comparison strain. *S. salivarius* were cultured and maintained with trypticase soy broth (BD Biosciences, USA) supplemented with 0.5% glucose (TSBG). *Porphyromonas gingivalis* ATCC 33277 was cultivated in brain heart infusion (BD Biosciences) supplemented with hemin (1 μg/ml) and vitamin K (0.2 μg/ml), and *Tannerella forsythia* ATCC 43037 was cultured modified new oral spirochete medium [17]. Finally, *Filifactor alocis* ATCC 35896 was cultivated using columbia media (BD Biosciences), L-cysteine·HCl (2 g/l), L-arginine·HCl (4 g/l), and yeast extract (5 g/l), hemin (1 μg/ml) and vitamin K (0.2 μg/ml). The periodontopathogens was maintained in anaerobic chamber (85% N_2_, 10% CO_2_, and 5% H_2_).

### Preparation of Surface Proteins from Bacteria

In order to examine effect of bacterial surface proteins on the induction of cytokine expression in THP-1 cells, *S. salivarius* were cultured in TSBG and harvested by centrifugation at 4,000 ×*g* for 5 min at 4°C. The bacteria were washed three times with cold phosphate buffered saline (PBS, pH 7.2) and resuspended in 1 ml of 8 M (Tris-buffered) urea containing 5 mM ethylene-diamine tetraacetic acid (EDTA) [18]. After incubation for 30 min at room temperature with shaking using a rotator (IKA Co., Germany), the supernatant was centrifuged at 11,000 ×*g* for 6 min to collect solubilized proteins. The solution containing solubilized proteins was filtered with 0.45 μm of polyvinylidene fluoride (PVDF) filter (Millipore, USA). The solution was transferred into standard RC dialysis membrane tubing (Spectrum Laboratories Inc., USA), and the tube was dialyzed in cold PBS (pH 7.2) to remove urea. The proteins were measured with Bradford assay kit (Bio-Rad Lab., USA).

### Preparation of LTA and LPS

LTA was extracted from *S. salivarius* and *F. alocis* according to method reported by Yoo and Lee [5]. The bacteria were collected by centrifugation at 7,000 ×*g* for 10 min at 4°C and washed three times with 0.1 M sodium citrate buffer (pH 4.5) followed by resuspended with 0.1 M sodium citrate buffer. The suspension was treated with lysozyme (100 μg/ml; Sigma-aldrich Co., USA) at 37°C for 3 h and immediately treated proteinase K (100 μg/ml; Promega Co., USA) at 55°C for 2 h. After performing ultra-sonication (Sonics & Materials Inc., USA) by cycling 20 × 10 sec pulses on ice output setting 10 W with 5 sec rests in between pulses, the suspension was added with an equal volume of n-butyl alcohol, and the mixture was then shaken on an orbital shaker for 2 h at room temperature followed by centrifugated at 13,000 ×*g* for 15 min, the upper phase was removed by suction, and the lower phase was transferred into standard RC dialysis membrane tubing (Spectrum Laboratories Inc., USA) in 0.1 M sodium citate with 15% *n*-propyl alcohol for 12 h to solubilize LTA. The preparation was reacted with octyl-sepharose CL-4B Fast flow (GE Healthcare Life Sciences, Sweden), and LTA was eluted with a linear gradient (15 to 60% of *n*-propyl alcohol in 0.1 M sodium citrate) in ion-exchange chromatography with DEAE-Sepharose (GE Healthcare Life Sciences, USA). Each fraction (3 ml) was investigated inorganic phosphate assay with ammonium molybdate to check containing LTA, and the fractions containing LTA were pooled and lyophilized. In other experiment, LPS was isolated from *P. gingivalis* and *T. forsythia* by the method of described by Lee SH. The bacteria were harvested by centrifugation at 7,000 ×*g* for 5 min at 4°C and washed two times with phosphate buffered saline (PBS, pH 7.2). The bacterial pellet was lysed with lysis buffer composed with phenol, DNase, and RNase. The mixture was vortexed until the pellet disappeared, and chloroform was then added into the mixture. After vortexing the mixture for 10 sec, the preparation was centrifuged at 13,000 ×*g* for 15 min at 4°C, and the supernatant was transferred into new tube. The supernatant was incubated with endonuclease (100 units/ml) (Merck Millipore, Germany) at 37°C for 2 h and treated with proteinase K (200 μg/ml) (Promega Co.) at 55°C for 1 h. the preparation was mixed with lysis buffer and chloroform flowing the protocol described above. The supernatant was mixed with isopropyl alcohol, and the LPS was harvested by centrifugation 12,000 × g for 15 min and washed with 75%ethyl alcohol. LPS was dissolved with endotoxin-free water and frozen at -80°C. After lyophilizing, the dry weight of LPS was measured.

### Labelling of LPS and LTA with Fluorescence and Biotin

The extracted LPS (1 mg) from *P. gingivalis* and *T. forsythia* or the extracted LTA (1 mg) from *F. alocis* were reacted with cold sodium metaperiodate at 4°C for 30 min to oxidize of polysaccharide part of LPS and LTA and then dialyzed in 100 mM sodium acetate (pH 5.5) for 12 h. The solution containing the LPS or the LTA was mixed with 10 mM Alexa Fluor 488 hydrizide (Invitrogen, USA) which was prepared by dissolving with 200 mM KCl and dialyzed two times in coupling buffer (0.1M sodium phosphate, 0.15M NaCl, pH 7.2) for 6 h followed by dialyzed three times in 1 L of endotoxin-free water for 12 h. After freezen drying the samples, the Fluor 488-labelled LPS and LTA was dissolved with endotoxin-free water at a concentration of 1 mg/ml. The fluorescence-labelled LPS was transferred into new tube and stored in -20°C.

### Cell culture and Treatment

THP-1 cells as a human monocytic cell line were purchased from the Amedrican Type Culture Collection and maintained with RPMI 1640 medium (Welgene, Korea) supplemented with 10% fetal bovine serum (Hyclone, USA) and antibiotics (100 U/ml of penicillin and 100 μg/ml of streptomycin sulfate) (Welgene). The cells were cultivated overnight, plated in 12-well plate (SPL Biosciences, Korea) at a concentration of 1 × 10^6^ cells/ml and treated with the surface extract from *S. salivarius* in the presence of heat inactivated human serum (Sigma-aldrich Co., USA). In other examination, the cells were treated with the surface extract from *S. salivarius* in the presence or the absence of *P. gingivalis* LPS, *T. forsythia* LPS, and *F. alocis* LTA. THP-1 cells were harvested to analyze signaling pathway using immune blot and RNA expression using real-time RT-PCR, and the culture supernatants were collected for ELISA and stored at -70°C until measuring cytokine production.

### Real-Time Polymerase Chain Reaction

The THP-1 cells were harvested by centrifugation at 2,000 ×*g* for 5 min at 4°C and treated with TRIzol RNA isolation kit (Invitrogen Life Tech., USA) to extract total RNA followed by performed the protocol according to the manufacturer’s method. Complementary DNA was synthesized by Maxime RT Premix (iNtRON, Korea). cDNA was mixed with 10 μl of 2× TB Green Premix EX Taq II (Takara Co., Japan), 0.4 μM of each specific primer, and ROX II dye. Distilled water was then filled to a final volume of 20 μl. The mixture was performed 40 PCR cycles (95°C for 15 sec, 60°C for 10 sec and 72°C for 33 sec) using ABI PRISM 7500 real-time PCR system (Applied Biosystems, USA). The amplification products were analyzed using a dissociation curve to confirm specific amplification. Critical threshold cycle (Ct) was defined as the cycle at which fluorescence became detectable as against the background and inversely proportional to the logarithm of the initial number of template cDNA. The levels of glyseraldehyde-3-phophate dehydrogenase (GAPDH) was used as a reference to normalize expression levels and quantify change of inflammatory cytokines. The sequences of primers for real-time PCR were as follows: 5¢-CAG GGA CCT CTC TCT AAT CA-3¢ and 5¢-AGC TGG TTA TCT CTC AGC TC-3¢ for the TNF-α gene; 5¢-GTG AAG GTG CAG TTT TGC CA-3¢ and 5¢-TCT CCA CAA CCC TCT GCA C-3¢ for the IL-8 gene; 5¢-GTG GTG GAC CTG ACC TGC-3¢ and 5¢-TGA GCT TGA CAA AGT GGT CG-3¢ for the GAPDH gene.

### ELISA

The conditioned media of various molecules treated cells were centrifuged at 7,000 ×*g* for 10 min at 4°C to remove cell debris, and the supernatants were transferred into new tube. TNF-α and IL-8 levels was measured by an ELISA kit (R&D systems, Inc., USA) according to the manufacturer’s protocol.

### LPS and LTA Binding Assay

To investigate LPS and LTA binding on the cell, THP-1 cells were treated with Alexa Fluor 488-labelled LPS or LTA in the presence or the absence of LTA from *S. salivarius* at the various concentrations at 37°C for 1 h in a CO2 incubator. The cells were harvested by centrifugation at 500 ×*g* for 3 min and washed three times with cold DPBS (Dulbecco’s phosphate buffered saline, pH 7.2). The binding of Alexa 488-labelled LPS and LTA to cells were analyzed by flow cytometry (FACS calibur, BD Biosciences, USA). The data were collected by counting 10,000 cells, and binding of Alexa Fluor 488-labeled LPS and LTA was detected in FL-1 channel. The data were analyzed using CellQuest software (BD Biosciences).

### Biding Assay to LBP and CD14

Anti-human LBP monoclonal Ab (MAB870, R&D Systems, USA) and anti-human CD14 polyclonal Ab (AF982, R&D systems) were dissolved in DPBS at a concentration of 1 μg/ml. The antibodies (50 ng/well) were coated on EIA plate (Corning Co., USA) at 4°C for 12 h. The antibody coated wells were washed five times with DPBS containing 0.1% tween 20 (DPBST) and blocked with 1% bovine serum album (BSA) in DPBST for 2 h in mild shaking condition. Recombinant human LBP (rhLBP, R&D Systems) and human CD14 (rhCD14, R&D Systems) were dissolved in DPBS at a concentration of 2 μg/ml and were added into suitable Ab-coated well. The plate was incubated at room temperature for 4 h and then washed five times with DPBST. The biotin-labelled LPS or LTA was treated or co-treated with *S. salivarius* LTA for 2 h at room temperature. Biotin-labelled LPS and LTA bound on rhLBP and rhCD14 were reacted with 50 μl of horseradish peroxidase (HRP)-labelled streptavidin (1 μg/ml) in DPBST containing 2% BSA for 1 h. After washing five times with DPBST, 3,3’,5,5’-tetramethylbenzidine (TMB) solution was added into the well. The plate was incubated at room temperature for 30 min, and the enzyme reaction was stopped by 1N sulfuric acid. The absorbance was measured at 450 nm of wavelength by a microplate reader (Biotek, USA).

### Statistical Analysis

Statistical analysis was performed by IBM SPSS statics ver. 23 software (IBM Co., USA). The data distribution was examined by Kolmogorov-Smirnov test, and the difference among groups of the data was analyzed by non-parametric Kruskal-Wallis test. Statistical significance among groups was defined by *p* value of less than 0.05. Post-hoc analysis to compare differences between individual groups was examined by Mann-Whitney U test, which was corrected for multiple comparisons by Bonferroni methods.

## Results

### Virulence of Surface Molecules of *S. salivarius*

**Fig. 1:**
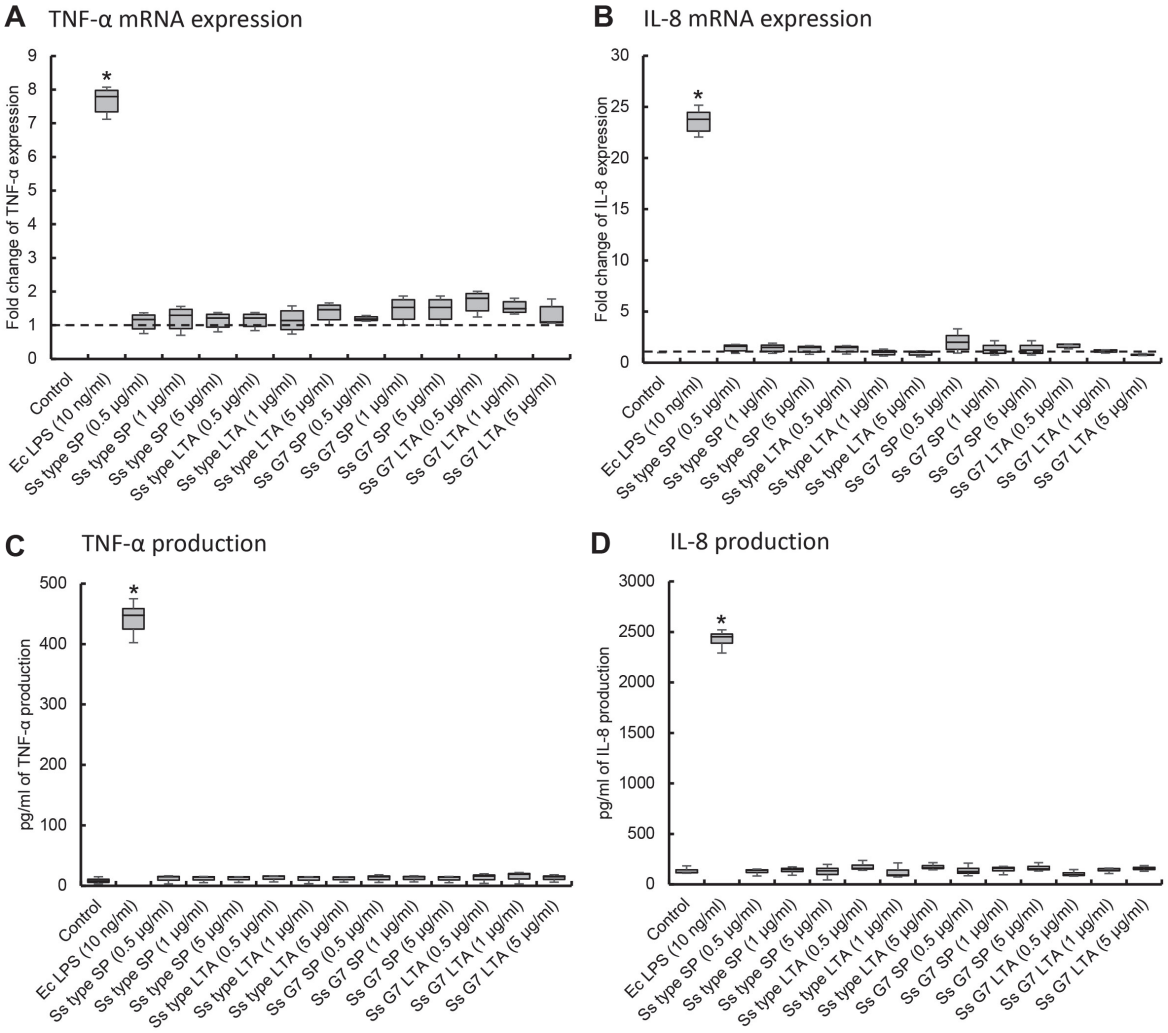
The effect of surface protein and LTA of *S. salivarius* on the induction of TNF-α and IL-8 expression. THP-1 cells were treated with the surface protein (SP) (0.5, 1, and 5 μg/ml) and LTA (0.5, 1, and 5 μg/ml) of *S. salivarius* or *E. coli* LPS (10 ng/ml). The expression of TNF-α (**A, C**) and IL-8 (**B, D**) was analyzed by real-time RT-PCR (**A, B**) and ELISA (**C, D**). The experiments were performed three times in triplicate, and data are represented as the median (horizontal lines), interquartile range (boxes), and full ranges (whiskers). Asterisk (*) indicates statistically significant differences compared with the control group (*p* < 0.05). The dotted line indicates the control level.

First, in order to investigate the virulence of surface molecules of *S. salivarius*, the surface proteins and the LTA were used. When The surface protein and LTA of *S. salivarius* type strain and G7 were treated on THP-1 cells at concentration of 0.5, 1 and 5 μg/ml, the two molecules showed no significant difference in the induction of TNF-α and IL-8 expression compared to the control (Figs. 1A and 1B). Furthermore, the surface protein and the LTA did not show significant difference in the induction of TNF-α and IL-8 production (Figs. 1C and 1D). *E. coli* LPS (10 ng/ml) as a positive control significantly induced TNF-α and IL-8 expression.

### Antagonistic Effect of *S. salivarius* LTA on LPS of Periodontopathogens

**Fig. 2:**
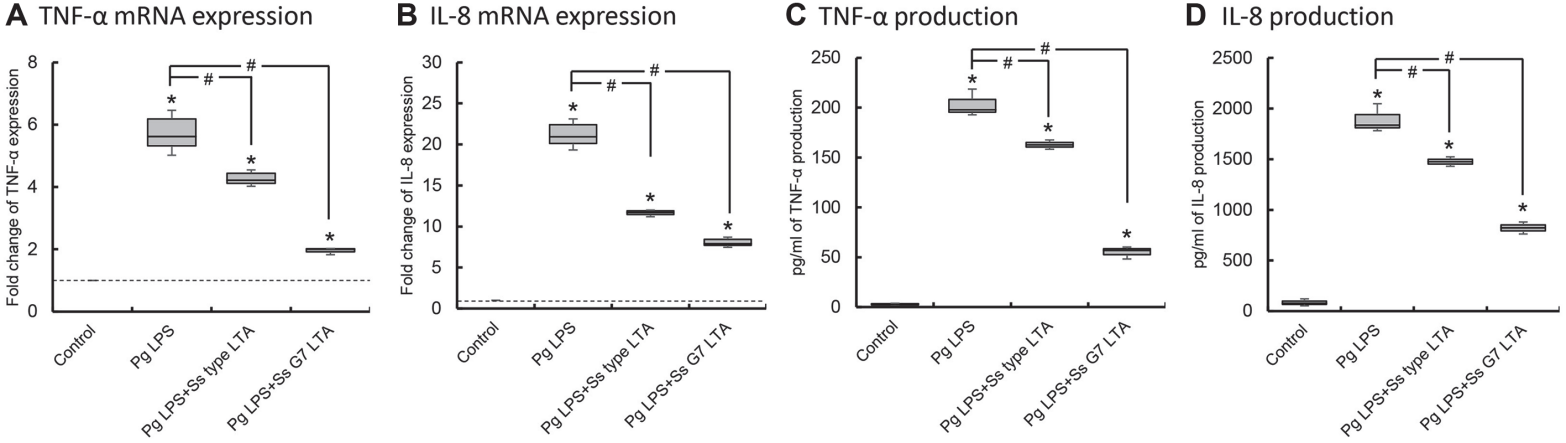
The inhibitory effect of LTA of *S. salivarius* on the induction of TNF-α and IL-8 expression by *P. gingivalis* LPS. THP-1 cells were co-treated with *S. salivarius* LTA (1 μg/ml) and *P. gingivalis* LPS (100 ng/ml), and the expression of TNF-α (**A, C**) and IL-8 (**B, D**) was measured by real-time RT-PCR (**A, B**) and ELISA (**C, D**). The experiments were performed three times in triplicate, and Asterisk (*) indicates statistically significant differences compared with the control group (*p* < 0.05). Sharp (#) indicates statistically significant differences compared with *P. gingivalis* LPS treated cell (*p* < 0.017). Data are represented as the median (horizontal lines), interquartile range (boxes), and full ranges (whiskers). The dotted line indicates the control level.

**Fig. 3:**
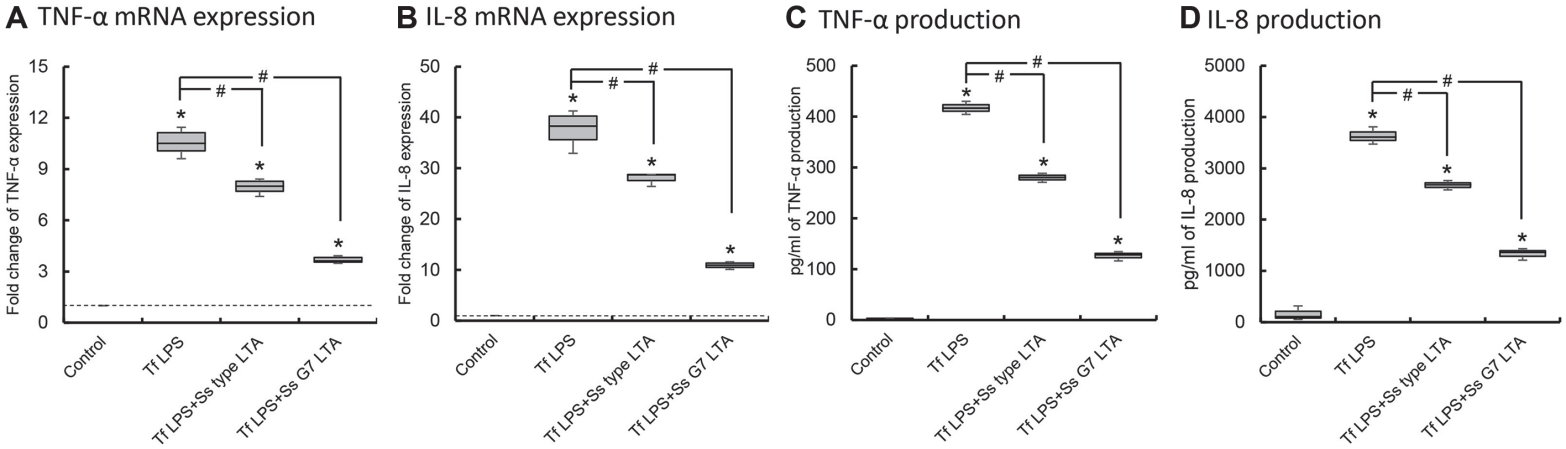
The inhibitory effect of LTA of *S. salivarius* on the induction of TNF-α and IL-8 expression by *T. forsythia* LPS. THP-1 cells were co-treated with *S. salivarius* LTA (1 μg/ml) and *T. forsythia* LPS (100 ng/ml), and the expression of TNF-α (**A, C**) and IL-8 (**B, D**) was measured by real-time RT-PCR (**A, B**) and ELISA (**C, D**). The experiments were performed three times in triplicate, and Asterisk (*) indicates statistically significant differences compared with the control group (*p* < 0.05). Sharp (#) indicates statistically significant differences compared with *T. forsythia* LPS treated cell (*p* < 0.017). Data are represented as the median (horizontal lines), interquartile range (boxes), and full ranges (whiskers). The dotted line indicates the control level.

The surface proteins from *S. salivarius* did not affect induction of LPS and LTA of periodontopathogens (data not shown). However, the LTA of *S. salivarius* showed inhibitory effect on the induction of pro-inflammatory cytokines. *P. gingivalis* LPS induced the expression of cytokines as TNF-α and IL-8, and *S. salivarius* LTA significantly inhibited the induction of TNF-α and IL-8 expression (Figs. 2 and S1). Also, LTA of *S. salivarius* type strain and G7 significantly reduced the cytokine expression induced by *T. forsythia* LPS (Figs. 3 and S2). LTA of *S. salivarius* G7 inhibited the expression of the cytokines induced by *P. gingivalis* and *T. forsythia* LPS more than LTA of *S. salivarius* type strain.

### Antagonistic Effect of *S. salivarius* LTA on LTA of *F. alocis*

**Fig. 4:**
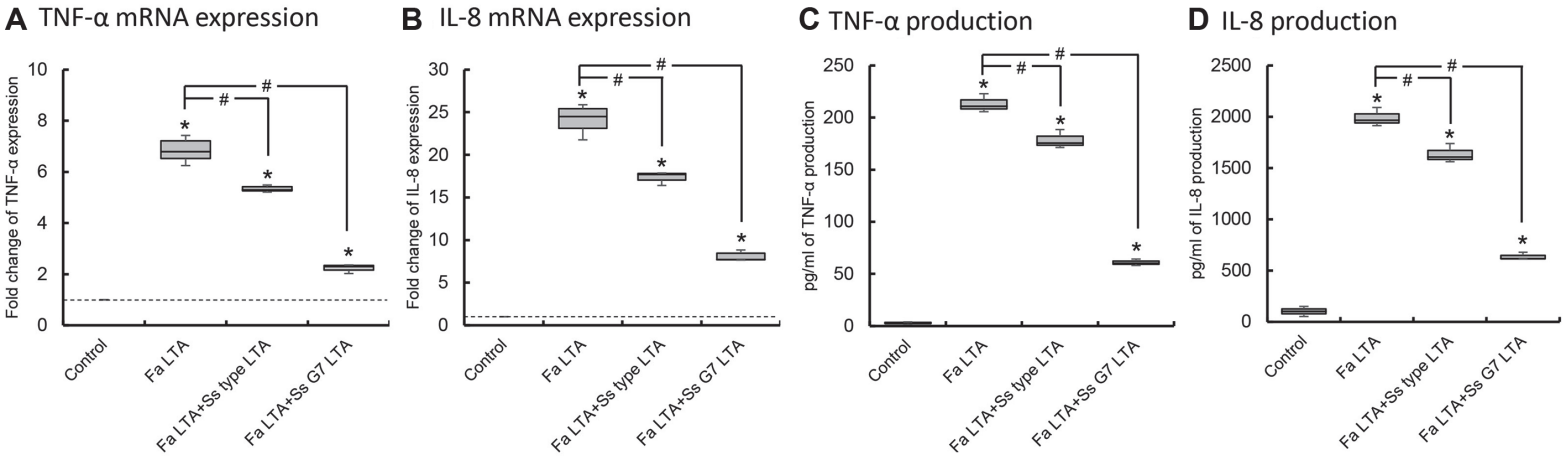
The inhibitory effect of LTA of *S. salivarius* on the induction of TNF-α and IL-8 expression by *F. alocis* LTA. THP-1 cells were co-treated with *S. salivarius* LTA (1 μg/ml) and *F. alocis* LTA (100 ng/ml), and the expression of TNF-α (**A, C**) and IL-8 (**B, D**) was measured by real-time RT-PCR (**A, B**) and ELISA (**C, D**). The experiments were performed three times in triplicate, and Asterisk (*) indicates statistically significant differences compared with the control group (*p* < 0.05). Sharp (#) indicates statistically significant differences compared with *F. alocis* LTA treated cell (*p* < 0.017). Data are represented as the median (horizontal lines), interquartile range (boxes), and full ranges (whiskers). The dotted line indicates the control level.

LTA of *F. alocis* as a periodontopathogen significantly induced TNF-α and IL-8 expression and production. As shown Figs. 4 and S3, LTA of *S. salivarius* significantly reduced TNF-α and IL-8 expression induced by LTA of *F. alocis*. Comparing two LTA of *S. salivarius*, LTA of G7 strain reduced the expression of TNF-α and IL-8 expression induced by *F. alocis* LTA more than LTA of type strain.

### Binding Inhibition of *S. salivarius* LTA

**Fig. 5:**
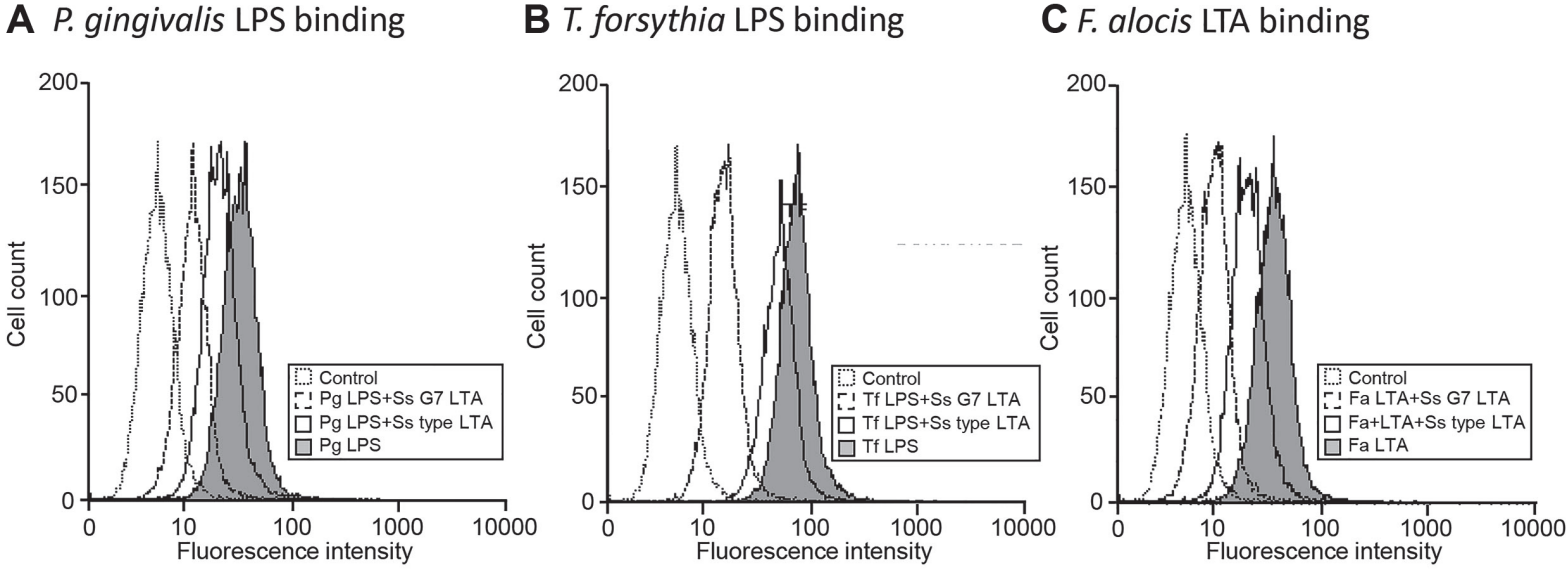
Binding inhibition of LPS or LTA of periodontopathogens by *S. salivarius* LTA. THP-1 cells were incubated with Alexa Fluor 488-labelled LPS (100 ng/ml) or LTA (100 ng/ml) of periodontopathogens alone or with *S. salivarius* LTA (1 μg/ml) for 1 h. The bound LPS or LTA was analyzed by flow cytometry.

In order to investigate inhibitory mechanism of *S. salivarius* LTA, LPS and LTA reactions occurring outer cells were investigated. After labelling LPS and LTA from periodontopathogens with fluorescence, the labelled LPS and LTA were cotreated on THP-1 cells with *S. salivarius* LTA. The levels of *P. gingivalis* and *T. forsythia* LPS binding to the cells was suppressed in the presence of *S. salivarius* LTA (Figs. 5A and 5B). Binding of *F. alocis* LTA on the cells was also reduced by LTA of *S. salivarius* G7 and type strain (Fig. 5C). Consistent with the cytokine inhibition tendency, LTA of *S. salivarius* G7 reduced the binding of LPS and LTA of periodontopathogens more than LTA of *S. salivarius* type strain.

### Involvement of CD14 and LBP in Antagonistic Effect of *S. salivarius* LTA

**Fig. 6:**
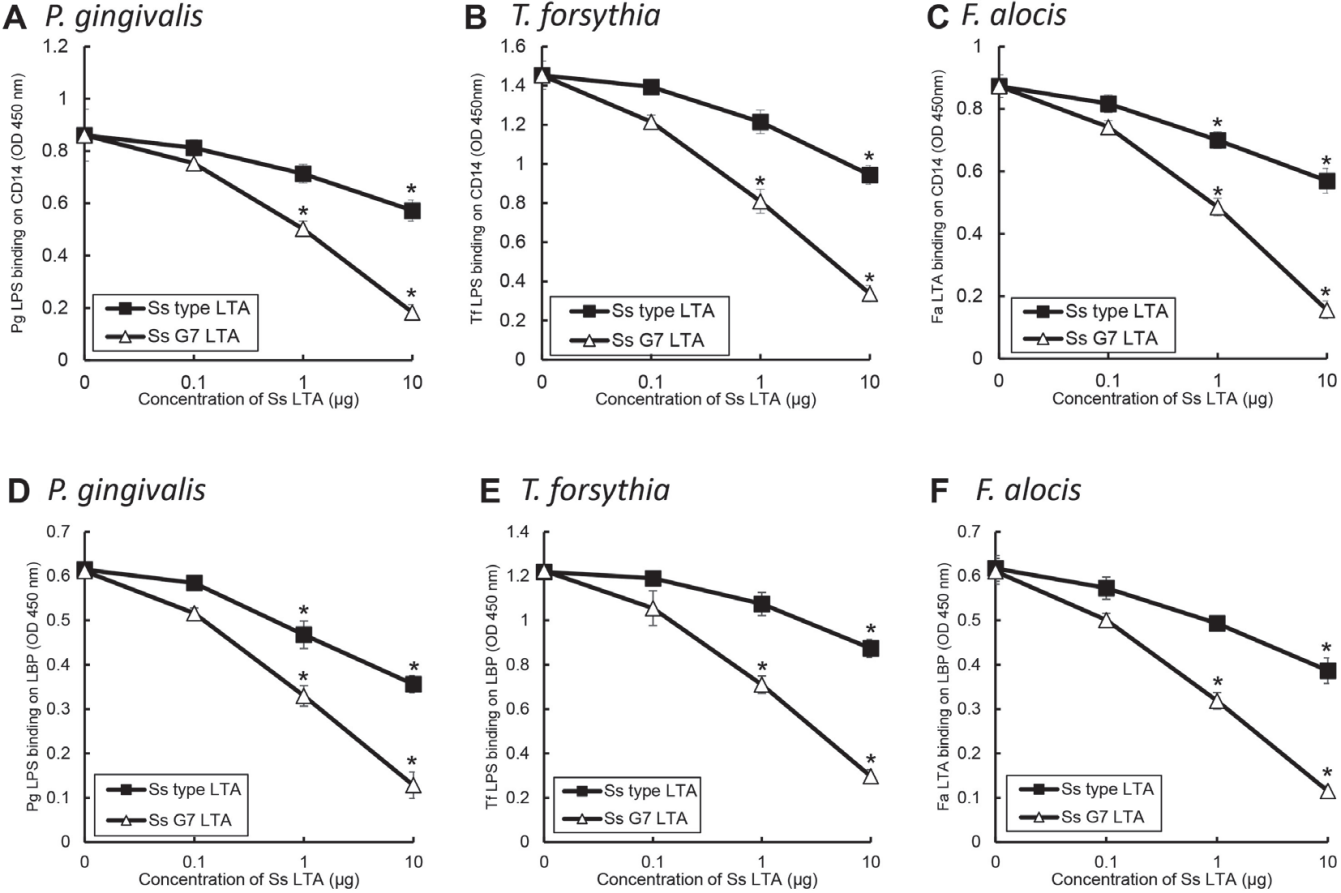
Binding inhibition of LPS or LTA of periodontopathogens to CD14 and LBP by *S. salivarius* LTA. EIA plates were coated with rhCD14 (A-C) and rhLBP (D-F). The plates were incubated with biotinylated *P. gingivalis* LPS (100 ng/ml), *T. forsythia* LPS (100 ng/ml), and *F. alocis* LTA (100 ng/ml) in the presence or the absence of *S. salivarius* G7 LTA and type strain LTA. The bound LPS and LTA were detected using HRP-labelled streptavidin and TMB solution. The experiments were performed three times in duplicate, and data are represented as means and standard deviations. Asterisk (*) indicates statistically significant differences compared with non-treated group (*p* < 0.05).

CD14 and LBP are involved in the binding and stimulating of LPS and LTA to cells [5, 17]. Therefore, we investigated whether the antagonistic effect of *S. salivarius* LTA was related to CD14 and LBP. *S. salivarius* LTA inhibited the binding of *P. gingivalis* and *T. forsythia* LPS to both CD14 and LBP (Fig. 6). Also, the LTA interrupted the binding of *F. alocis* LTA to CD14 as well as LBP.

## Discussion

The oral cavity is characterized by the presence of multi-species bacteria like gut tract and the coexistence of soft and hard tissues unlike gut tract. Therefore, to apply beneficial bacteria for the oral cavity is more difficult than for colonic conditions. Until recently, probiotics were developed for the purpose of preventing oral diseases induced by bacteria, and some bacteria were recommended as beneficial bacteria and progressed to the commercialization stage [19-21]. However, among these probiotics, some species have aciduricity like *Streptococcus mutans* which is a cariogenic bacterium and can have harmful effects on the tooth as a hard tissue [22, 23]. Therefore, we began to investigate the beneficial effects of isolated *S. salivarius*, which do not have aciduricity and is a commensal bacterium, and first investigated the inhibitory effect on inflammation in this study.

The surface molecules of *S. salivarius* G7, surface protein and LTA, did not induce the expression of inflammatory cytokines. The bacterial surface molecules are the components that attaches first to the cells and relates with biofilm formation. Therefore, the characteristics of *S. salivarius* surface molecules were first investigated and the effects of the surface molecules on LPS and LTA of periodontopathogens, which are periodontitis inducing factor, were investigated. When the LPS and the LTA of periodontopathogens was co-treated with the surface molecules of *S. salivarius* G7 on the cells, LTA of *S. salivarius* G7 inhibited the induction of inflammatory cytokine expression by LPS and LTA of periodontopathogens such as *P. gingivalis*, *T. forsythia*, and *F. alocis*. Surface protein of *S. salivarius* G7 did not affect. General LPS and LTA bind and stimulate Toll-like receptor (TLR) 4 and 2, respectively [24]. *T. forsythia* LPS activates human cell via TLR4, and *P. gingivalis* LPS stimulates human cell via TLR2 and TLR4 according to hemin concentration [17, 25]. Also, *F. alocis* LTA activates TLR2 [5]. Therefore, in order to analyze the mechanism of the inhibitory effects of *S. salivarius* G7 LTA on LPS and LTA of periodontopathogens, cell attachment of its LPS and LTA was first investigated. *S. salivarius* G7 LTA inhibited the binding of LPS and LTA of periodontopathogens to the cells. It is known that LTA stimulates cells by binding to TLR2 and TLR6 heterodimers [26]. If *S. salivarius* G7 LTA first bind to TLR2 first and inhibits the binding of other ligand to TLR2, the inhibition for activity of *T. forsythia* LPS to cell cannot occur. *Treponema medium* and *T. socaranskii* glycolipid has antagonistic activity against TLR4 ligand [27, 28], and *Capnocytophaga ochracea* and *P. gingivalis* LPS also showed the antagonism against *Escherichia coli* LPS as a TLR4 ligand [29]. As shown in these studies, most studies have been reported the antagonistic effect of the ligand for the same receptor, and it is not known that LTA has an inhibitory effect on both LPS and LTA as in this study. In addition, the binding inhibition of *S. salivarius* G7 LTA was not clearly observed without serum.

Finally, we evaluated whether the antagonistic effect of *S. salivarius* G7 LTA on LTA and LPS of periodontopathogens was related to CD14 and LBP. The action of LBP and CD14 is required for LPS and LTA to bind to and stimulate TLRs [24]. LPS and LTA form micelle in aqueous solution due to hydrophilic (O-antigen of LPS and glycerol-phosphate unit of LTA) and hydrophobic unit (lipid A for LPS and diacyl chain of LTA) [17, 30]. Micellized LPS and LTA is transported by LBP to CD14 which concentrates LPS and LTA on TLR4 and TLR2, respectively [31, 32]. As shown in the results, *S. salivarius* G7 LTA inhibit LPS and LTA of periodontopathogens binding to LBP and CD14 in a dose-dependent manner. The structure of LTA describes diacylglycerol-containing glycolipid anchor and a covalently coupled polymeric backbone (called glycerol-phosphate units) [33], and the diacyl chains as fatty acid in LTA attaches TLR2 and TLR6 heterodimer [26]. Therefore, the structural different of diacyl chain indicate differences in bioactivity [34]. In detail, attachment and stimulation of the diacyl chain for TLR2 differ according to its number of carbon atoms, and depending on the structure of its glycerol-phosphate units, the binding to CD14 and LBP is different [35, 36]. Based on these studies, *S. salivarius* G7 LTA has the binding site for CD14 and LBP, but its acyl chain may not stimulate TLR2. Furthermore, *S. salivarius* G7 LTA bind and consume CD14 and LBP, thereby reducing the binding of LPS or LTA of periodontopathogens to CD14 and LBP. Eventually, the transfer of LPS or LTA of periodontopathogens to TLR may be reduced. By these mechanisms, *S. salivarius* G7 LTA could inhibit periodontopathogen LPS or LTA-induced inflammation.

Taken together, the surface molecules of *S. salivarius* G7 did not affect the induction of inflammatory cytokine expression. Among surface molecules, LTA of *S. salivarius* G7 showed inhibitory activity to inflammatory cytokines induced by LPS or LTA of periodontopathogens. Furthermore, these inhibitory effects may be caused by inhibiting the binding of LPS and LTA of periodontopathogens to CD14 and LBP, which are required for cell receptor attachment.

In conclusions, the surface molecules of *S. salivarius* G7 did not induce the expression of inflammatory cytokines, and LTA of *S. salivarius* G7 inhibited the expression of inflammatory cytokines induced by LPS of *P. gingivalis* and *T. forsythia* or LTA of *F. alocis*. The anti-inflammatory mechanism of *S. salivarius* G7 LTA was demonstrated by its inhibitory effect on competition with the LPS and the LTA of periodontopathogens for attachment of CD14 and LBP. *S. salivarius* G7 may be a candidate for prevention of periodontitis induced by LPS and LTA of bacteria.

## Supplemental Materials

Supplementary data for this paper are available on-line only at http://jmb.or.kr.
