# Corrigendum to: Anti-Inflammatory Efficacy of Human-Derived *Streptococcus salivarius* on Periodontopathogen-Induced Inflammation

**DOI:** 10.4014/jmb.2023.3310.1402

**Published:** 2023-10-28

**Authors:** Dong-Heon Baek, Sung-Hoon Lee

**Affiliations:** Department of Oral Microbiology and Immunology, College of Dentistry, Dankook University, Cheonan 31116, Republic of Korea

In the article titled “**Anti-inflammatory Efficacy of Human-Derived *Streptococcus salivarius* on Periodontopathogen-induced Inflammation**”, the authors noticed that the affiliation was incorrect due to a typing error. The affiliation is “Department of Oral Microbiology and Immunology, College of Dentistry, Dankook University, Cheonan 31116, Republic of Korea”, not “Department of Dental Hygiene, College of Health Science, Dankook University”. The corrected affiliation is now available online.

The authors sincerely apologize for the confusion caused by this error and state that this does not change the scientific conclusion of the article in any way.

