# Supplementary material for: Anti-Inflammatory Efficacy of Human-Derived Streptococcus salivarius on Periodontopathogen-Induced Inflammation
Source: J Microbiol Biotechnol. 2023 May 11;33(8):998–1005. doi: 10.4014/jmb.2302.02002 (PMC10468666; doi:10.4014/jmb.2302.02002)
Supplement: Supplementary file 1 [file jmb-33-8-998-supple.pdf]

J. Microbiol. Biotechnol.
https://doi.org/10.4014/jmb.2302.02002

Supplementary Figures

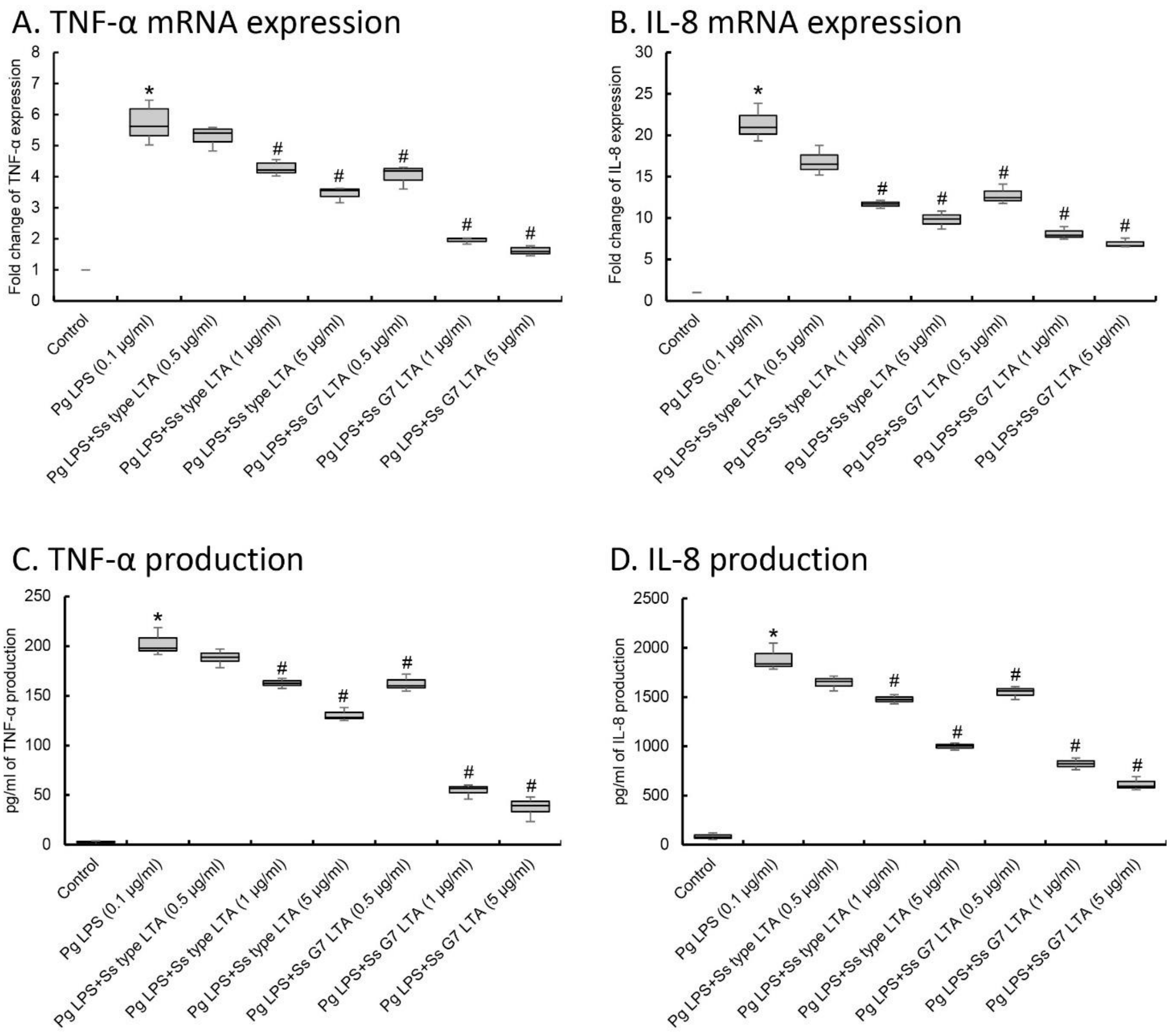

Supplementary Figure 1. The inhibitory effects of LTA of *S. salivarius* on the induction of TNF-α and IL-8 expression by *P. gingivalis* LPS. THP-1 cells were co-treated with *S. salivarius* LTA (0.5, 1 and 5 μg/ml) and *P. gingivalis* LPS (100 ng/ml), and the expression of TNF-α (A, C) and IL-8 (B, D) was measured by real-time RT-PCR (A, B) and ELISA (C, D). The experiments were performed three times in triplicate, and Asterisk (*) indicates statistically significant differences compared with the control group ($p < 0.05$). Sharp (#) indicates statistically significant differences compared with *P. gingivalis* LPS treated cell ($p<0.017$). Data are represented as the median (horizontal lines), interquartile range (boxes), and full ranges (whiskers). The dotted line indicates the control level.

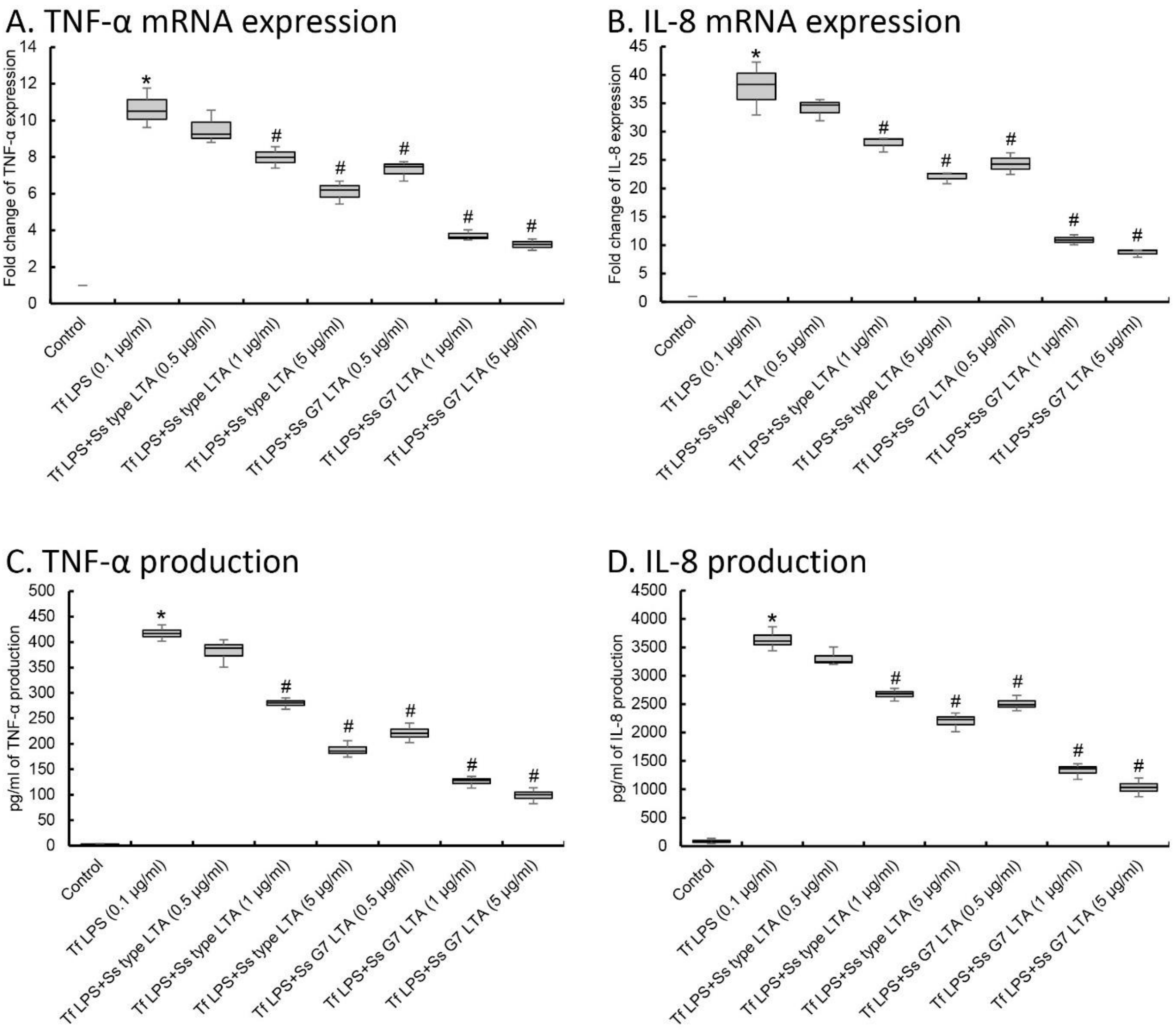

Supplementary Figure 2. The inhibitory effects of LTA of *S. salivarius* on the induction of TNF-α and IL-8 expression by *T. forsythia* LPS. THP-1 cells were co-treated with *S. salivarius* LTA (0.5, 1 and 5 μg/ml) and *T. forsythia* LPS (100 ng/ml), and the expression of TNF-α (A, C) and IL-8 (B, D) was measured by real-time RT-PCR (A, B) and ELISA (C, D). The experiments were performed three times in triplicate, and Asterisk (*) indicates statistically significant differences compared with the control group ($p < 0.05$). Sharp (#) indicates statistically significant differences compared with *T. forsythia* LPS treated cell ($p < 0.017$). Data are represented as the median (horizontal lines), interquartile range (boxes), and full ranges (whiskers). The dotted line indicates the control level.

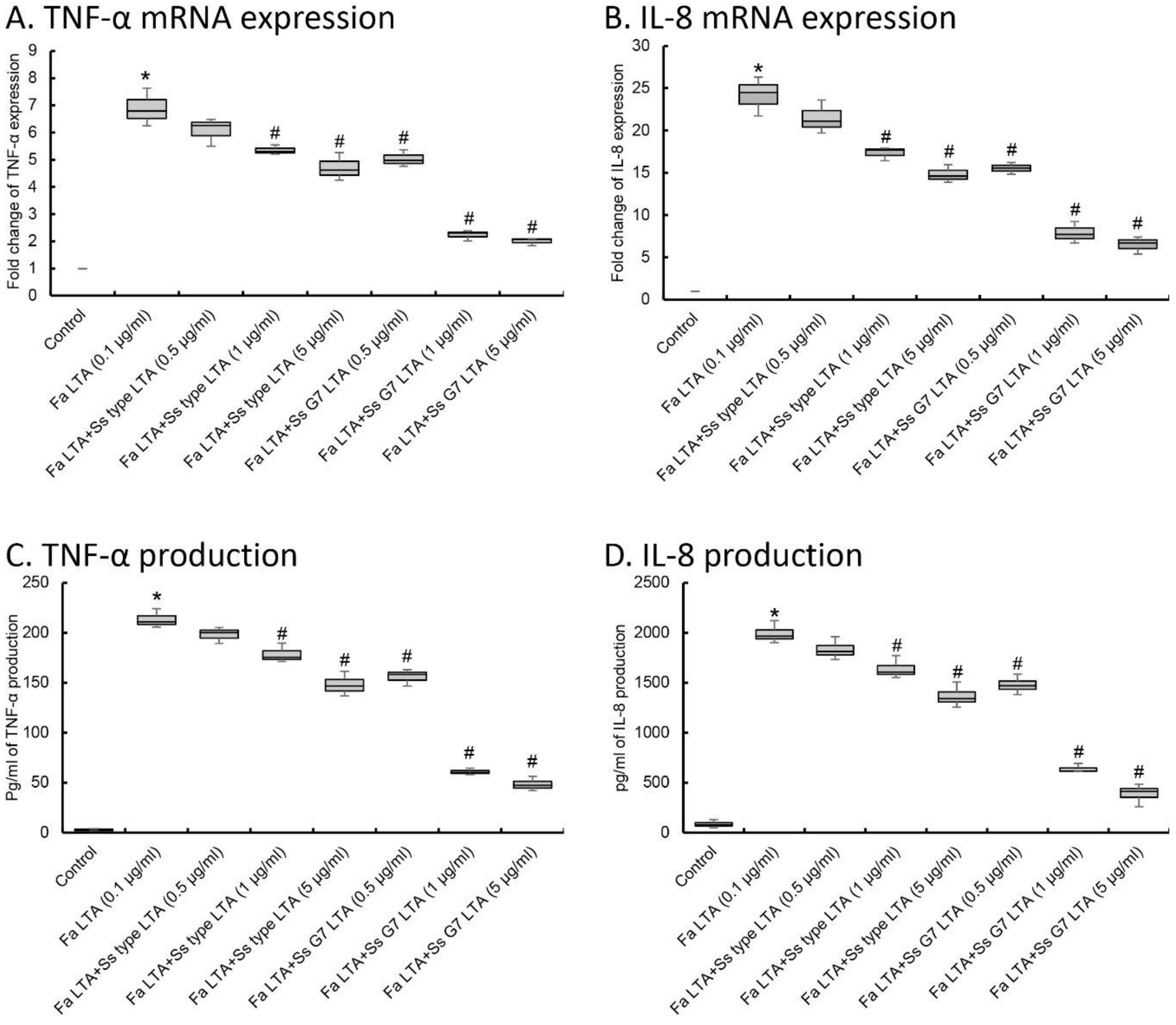

Supplementary Figure 3. The inhibitory effects of LTA of *S. salivarius* on the induction of TNF-α and IL-8 expression by *F. alocis* LTA. THP-1 cells were co-treated with *S. salivarius* LTA (0.5, 1 and 5 μg/ml) and *F. alocis* LTA (100 ng/ml), and the expression of TNF-α (A, C) and IL-8 (B, D) was measured by real-time RT-PCR (A, B) and ELISA (C, D). The experiments were performed three times in triplicate, and Asterisk (*) indicates statistically significant differences compared with the control group (p < 0.05). Sharp (#) indicates statistically significant differences compared with *F. alocis* LTA treated cell (p<0.017). Data are represented as the median (horizontal lines), interquartile range (boxes), and full ranges (whiskers). The dotted line indicates the control level.
